# The impact of long-term social housing on biconditional association task performance and neuron ensembles in the anterior cingulate cortex and the hippocampal CA3 region of aged rats

**DOI:** 10.18632/aging.206310

**Published:** 2025-08-22

**Authors:** Anne M. Dankert, Abbi R. Hernandez, Taylor B. Wise, Katrina I. T. Dayaw, Judith N. T. Dayaw, Rachael M. Layden, Katelyn N. Lubke, Sara N. Burke, Victoria L. Templer

**Affiliations:** 1 Department of Psychology, Providence College, Providence, RI 02918, USA; 2 Department of Psychology and Neuroscience, University of North Carolina, Chapel Hill, NC 27514, USA; 3 Department of Medicine and Gerontology, University of Alabama at Birmingham, Geriatrics and Palliative Care, Birmingham, AL 35294, USA; 4 Department of Cognitive, Linguistic, and Psychological Science, Brown University, Providence, RI 02912, USA; 5 Department of Neuroscience, University of Florida, Gainesville, FL 32611, USA

**Keywords:** aging, environmental enrichment, working memory, complex cognition, immediate early genes

## Abstract

Cognitive decline and changes in neuronal activity are hallmarks of aging. While environmental enrichment (EE) can protect against cognitive deficits in old age, whether EE with long-term social housing provides greater protection than EE alone, and the underlying neuronal mechanisms, remain unknown. Here, aged socially housed (SH), aged non-socially housed (NSH), and young rats were tested on the biconditional association task (BAT), a test of cognitive flexibility in which the rewarded object depends on the subjects’ location in a Y maze. Immediate early genes were used to assess neuronal activity during BAT performance and a working memory alternation task in which rats traversed the arms of the Y maze but were not required to select the correct object on either side of the maze. NSH rats had significantly impaired working memory compared to SH rats and significantly impaired performance on BAT compared to both young and SH rats, indicating that social housing protects cognitive flexibility during aging beyond EE alone. SH rats displayed greater CA3 hippocampal activity during BAT, and lower anterior cingulate cortex activity during the alternation task compared to NSH rats, suggesting that neuronal activity differences in these regions may explain preserved cognition in SH animals.

## INTRODUCTION

Cognitive decline and changes in neuronal activity are hallmarks of advancing age across species. It is well established that several cognitive domains, including memory and executive functioning, show age-related impairments [1–7]. While life expectancy is increasing, most people over the age of 65 will experience cognitive decline. Although mild cognitive impairment and dementia are prevalent in older populations, cognitive deficits are also commonly observed in elderly individuals with no neurodegenerative diseases. These cognitive deficits greatly reduce the quality of life for affected individuals and are predictive of negative patient outcomes. Thus, it is essential to discover ways to inhibit the progression of cognitive decline due to aging.

Among the first brain regions to show age-associated dysfunction in both humans and rats are the prefrontal cortex (PFC), including the anterior cingulate cortex (ACC), and the medial temporal lobe (MTL), including the hippocampus and perirhinal cortex (PER) [8–10]. However, rather than mere neuronal cell loss in these regions, age-associated dysfunctions are often due to changes in neuronal activity patterns [1, 5, 7, 9, 11–16]. Superagers, defined as older adults with memory and cognitive abilities similar to young adults, have shown both increased cortical thickness in the ACC and stronger functional connectivity with regions in the salience network compared to normal agers [17, 18]. In aged rats with behavioral impairments, the ACC has also been shown to have enhanced functional connectivity specifically between the dorsal striatum and deep layers of the ACC compared to young rats [19]. Additionally, many memory impairments observed in old age correlate with morphological changes in both the CA1 and CA3 regions of the hippocampus [20]. Aging is correlated with the loss of neurons in the CA1 region, particularly pyramidal neurons, which affects the integrity of hippocampal circuitry. In addition to neuronal loss, dendritic arborization reduction, and alterations in glial cells, studies have demonstrated that older adults experience a decline in both the quantity and efficacy of synaptic connections in the CA1 region, potentially contributing to memory impairments. These structural changes are accompanied by functional modifications in long-term potentiation (LTP), gene expression, calcium regulation, and neurotransmitter release, especially gamma-aminobutyric acid (GABA) [21]. Similarly, the CA3 region also shows structural and functional alterations with aging including volumetric loss, neuronal and synaptic loss, and morphological changes in synapses [11, 22–27]. While the degree of neuronal loss in CA3 is often less pronounced than in CA1, various studies have found age related deficits due to altered neuronal activity in CA3. Specifically, CA3 is implicated in pattern separation and completion, and is associated with working memory deficits due to structural changes believed to affect the retrieval and manipulation of information [20].

Behaviors that rely on these structures are also vulnerable to decline with advancing age. For example, it is widely accepted that working memory impairments in old age are linked to dysfunction of the PFC [1, 2, 6] and CA3 [20]. One study found that working memory measured through a delayed alternation task was impaired in aged rats compared to young, and that this impairment could be reversed with a D1 receptor agonist in the PFC [28]. In addition to impaired working memory, cognitive flexibility can also decline with age. Aged rats are consistently impaired on the Biconditional Association Task (BAT; also known as the Object-Place Paired Association (OPPA) Task), which requires rats to learn that the target object in a pairwise discrimination problem changes based on its location within the maze [29–32]. Rats must use distinct allocentric cues in each arm of a Y maze to displace the correct object, where object A is correct in one arm while object B is correct in the other arm. A previous study found that rats with lesions to the CA3, but not CA1, were significantly impaired at learning object-place paired associations [33]. BAT not only relies on use of the hippocampus and PFC but requires functional connectivity between multiple brain regions including the hippocampus, PFC, and PER [32, 34, 35].

While the hippocampus and its adjacent structures and PFC are affected by aging, interventions targeting these areas have shown promise in mitigating cognitive decline. Pharmacological and dietary interventions, such as anti-inflammatory compounds, have been explored to target age-related changes in these regions. Environmental enrichment (EE), including physical exercise, cognitive stimulation, and social interaction are also becoming increasingly well-known to combat cognitive decline. Though it is well established that EE such as exercise has positive effects on neuronal integrity and associated cognitive behaviors, especially in old age [36–54], the independent effect of sociality and the mechanisms by which it may protect memory performance and neuronal changes such as synaptic plasticity and neurogenesis, is far from established [55–60]. First, most studies that evaluate EE do not separate other forms of EE such as exercise from social enrichment. Second, social interaction effects on cognition are often studied with complete social isolation rather than mirroring non-social behavior in humans. Rats share many of the memory and executive functioning deficits seen in human aging [8, 9, 12, 20, 61–68]. Rats are also social animals [69–71] and have a relatively short lifespan - approximately three years - which makes them ideal for longitudinal studies of aging and for investigating the neuronal mechanisms of age-associated cognitive decline. Additionally, whether long-term social housing can protect against age associated impairments on BAT, and whether these effects are related to altered neural activity is not known.

Here we investigate the differences between socially housed (SH) aged rats, non-socially housed (NSH) aged rats, and standard young rats in performance on BAT and a working memory alternation task and the corresponding neuronal activity in the CA3 and ACC using *Arc*/*Homer*1a double Compartmental Analysis of Temporal activation with Fluorescence *in situ* Hybridization (catFISH) [72–75]. While both the CA1 and CA3 subregions of the hippocampus are important for memory, previous studies have found that the CA3, but not the CA1, is necessary for learning object-place paired associations [33] and aged rats that show impaired BAT performance do not show differences in neuronal activity in the CA1 compared to young rats [31]. Therefore, the current study aims to identify whether altered neuronal activity in the CA3 region of the hippocampus may underlie impaired BAT performance in aged rats and whether social housing may attenuate this change in neuronal activity and prevent the age-related decline in cognitive flexibility. The current study also focuses on the ACC region of the PFC as the ACC is thought to play a central role in goal-directed behavior and memory [76], so differences in memory impairments between SH and NSH aged rats may occur in part due to effects of sociality on neurons in the ACC. The ACC has also been shown to be crucial for adapting behaviors based on environmental changes such as in reversal learning [77]. Interestingly, successful human agers that report more positive social relationships show morphological differences such as increased cortical thickness in the ACC than older adults with more cognitive difficulties [17, 78, 79]. The ACC and CA3 were segmented into superficial and deep and proximal and distal layers, respectively, as previous work has found that differences in neuronal activity during BAT between young and aged rodents depend on the specific layer of the region of interest [31]. Further, both the CA3 and ACC exhibit cytoarchitectural differences and differential patterns of connectivity depending on subregion [26, 76, 80, 81]. For example, the deeper layers of ACC (L5) are found to be more excitable than superficial layers (L3) and communicate with other regions like the amygdala which show a more limited dynamic range and act to refine signaling more locally [76].

All aged rats received enrichment, including handling, physical enrichment, opportunities for exercise, and prior cognitive testing. However, only the SH animals received social enrichment, as SH rats were group housed for their entire lives while NSH rats were always individually housed. Our primary hypothesis is that long-term social housing will slow the process of normal cognitive decline in the SH rats, as indicated by fewer working memory errors and a greater proportion of correct choices on BAT compared to NSH rats. Additionally, we hypothesized that SH rats would show altered neuronal activation in the CA3 and ACC during the BAT test compared to NSH rats, providing a potential neurological mechanism for the effect of sociality on cognitive decline due to aging. Further, based on the differences in layers of CA3 and other cortical regions discussed above, we assessed subregions of the CA3 and ACC to investigate whether effects of social housing on neuronal activity in our paradigm are subregion specific. Additionally, since a large quantity of neurobiological research is conducted on rodents with little environmental or social enrichment, the present study further compares the behavior and neuronal activity of the aged rats to that of a group of young, individually housed rats, which has historically been the standard in the field. Therefore, our secondary hypothesis is that NSH aged rats will show significant impairment on the BAT test compared to the standard young rats, while the SH aged rats will perform comparably to the standard young rats. Although previous research has demonstrated differences in BAT performance and changes in neuronal activity between young and aged rats [31, 32], to our knowledge, this is the first study to investigate the effect of longitudinal social versus non-social housing on BAT performance and neuronal activity in the CA3 and ACC.

## RESULTS

### Pre-training and initial response bias

**Figure 1:**
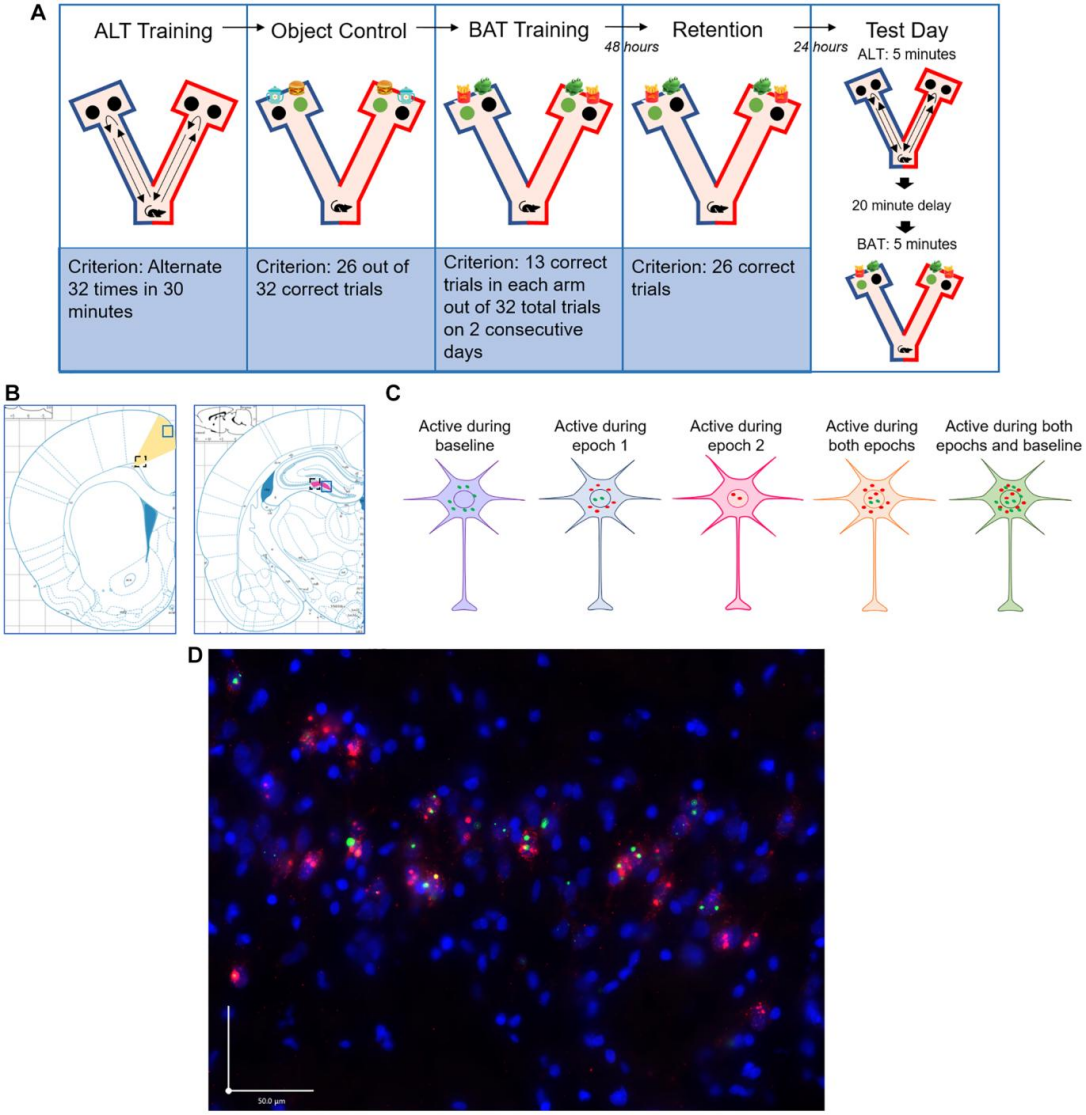
**Experimental methods.** (**A**) Timeline of BAT training and test day. Green circles represent correct choice; black circles represent incorrect choice. Object placement over the left and right food well was randomized and counterbalanced across trials. Test day epochs were counterbalanced across subjects so that half of the animals were tested on the alternation task (ALT) first and the other half were tested on the biconditional association task (BAT) first. (**B**) Brain regions imaged (ACC: yellow, CA3: pink). Black dashed line and blue solid line squares indicate deep and superficial areas of the ACC and distal and proximal areas of the CA3 respectively. (**C**) Immediate early gene cellular distribution for neurons that were active during baseline, during epoch 1 only, during epoch 2 only, during both epochs, and during both epochs and baseline. Red dots signify *Arc* expression and green dots signify *Homer*1a expression. (**D**) Representative microscopic image of subcellular distribution of *Arc* (red) and *Homer*1a (green) within neurons where the nuclei were counterstained with DAPI (blue). Scale bar is 50 μm.

All rats were trained and tested on BAT as well as a simple alternation task (ALT). During ALT, rats were required to alternate between the left and right arms of a Y maze to receive a food reward and traveling down the previously chosen arm of the maze was considered a working memory error (Figure 1A). For BAT, rats again had to alternate between the left and right arms of the maze, but receipt of the food reward depended on displacement of the correct object. Importantly, the displacement of one object was rewarded in the left arm while displacement of the other object was rewarded in the right arm (Figure 1A). This task is cognitively challenging and requires the rat to associate its location in the maze with the correct object.

**Figure 2:**
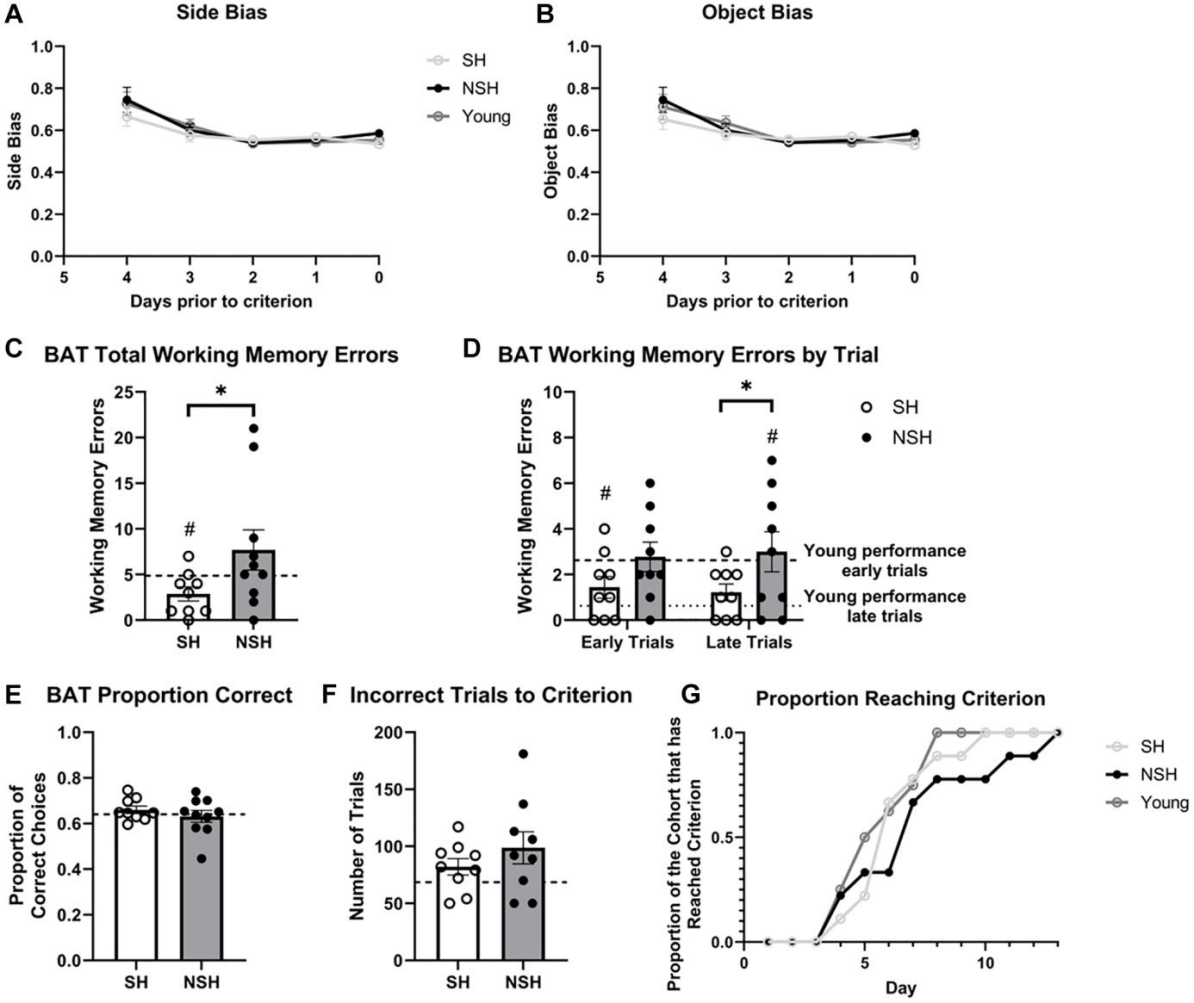
**Performance during BAT training.** No group differences in (**A**) side bias or (**B**) object bias during BAT training were observed. (**C**) Total number of working memory errors made during BAT training. (**D**) Number of working memory errors made during the first half (early trials) and second half (late trials) of each BAT training session. (**E**) Proportion of correct choices made during BAT training. (**F**) Number of incorrect trials prior to reaching criterion. (**G**) Proportion of the cohort reaching criterion across day of BAT training. Abbreviations: BAT: biconditional association task; SH: socially housed; NSH: non-socially housed. Error bars represent standard error of the mean. Dashed lines represent population (test-values) averages obtained from the young cohort and ^#^signifies *p* < 0.05 for these one sample *t*-tests. ^*^signifies *p* < 0.05 for two sample *t*-tests.

During the initial training phase of BAT, rats displayed a noticeable side bias, showing a preference for well location rather than the correct object (Figure 2A). As previously discussed by Hernandez et al. (2017), this bias is likely modulated by mPFC projections to sensorimotor regions [82]. A repeated measures analysis of variance (ANOVA) revealed that side biases diminished over time (main effect of day: F_(4,115)_ = 3.954, *p* = 0.0048, η^2^ = 0.115). However, there were no discernible differences between the groups (main effect of group: F_(2,115)_ = 2.856, *p* = 0.0616, η^2^ = 0.041), and no interaction was observed (F_(8,115)_ = 0.1554, *p* = 0.996, η^2^ = 0.009). Bonferroni comparisons demonstrated no significant distinctions between groups at any given day before reaching criterion (*p* > 0.05). Similarly, rats exhibited an object bias, favoring certain objects when they served as both the correct object (in one arm) and the incorrect object (in the other arm) (Figure 2B). The results paralleled those of the side bias, indicating a declining object bias over time, which occurred at an equivalent rate for all rats as determined by a repeated measures ANOVA (main effect of day: F_(4,115)_ = 13.74, *p* < 0.0001, η^2^ = 0.309; main effect of group: F_(2,115)_ = 1.001, *p* = 0.373, η^2^ = 0.011; day x group: F_(8,115)_ = 0.8065, *p* = 0.592, η^2^ = 0.036). Follow-up multiple comparisons using Bonferroni corrections showed no significant group differences on any of the test days (*p* > 0.05). Collectively, these findings imply that no strategy or persistent side bias was favored by any group over another. Instead, all rats, irrespective of their group assignment, exhibited reductions in response biases as they acquired proficiency in the task and met criterion.

### Non-socially housed aged rats demonstrate impaired working memory during BAT training compared to socially housed aged rats

A one-tailed independent samples *t*-test revealed that SH animals made significantly fewer working memory errors during BAT training than NSH animals (t_(11.50)_ = −2.058, *p* = 0.032, g = −0.690, Figure 2C). Because trials at the beginning of a session may rely more heavily on reference memory than working memory due to initial access of the long-term memory stores that indicate task parameters (e.g., “object C is correct on this side but not the other”) from the previous session, we further divided each training session into the first half of the trials (early trials) and the second half of the trials (late trials), as has been described previously [83, 84] to better isolate the working memory processes that predominate in later trials. While SH rats made fewer working memory errors than NSH rats during the early trials, this effect did not reach significance (t_(16)_ = −1.672, *p* = 0.057, d = −0.788, Figure 2D), However, SH animals did make significantly fewer working memory errors than the NSH animals during the late trails (t_(10.653)_ = −1.863, *p* = 0.045, d = 0.878, Figure 2D) indicating that long term social housing confers benefits to working memory in old age. No significant differences were observed between SH and NSH animals for proportion of correct choices during BAT training (t_(17)_ = 0.892, *p* = 0.192, g = 0.392, Figure 2E) or number of incorrect trials to criterion (t_(16)_ = −1.051, *p* = 0.155, d = −0.495, Figure 2F). These results signify that both groups learned the task at a similar rate. However, more days were required for the entire NSH group to reach criterion (Figure 2G).

### Non-socially housed aged rats were significantly impaired on the BAT test compared to socially housed aged rats

**Figure 3:**
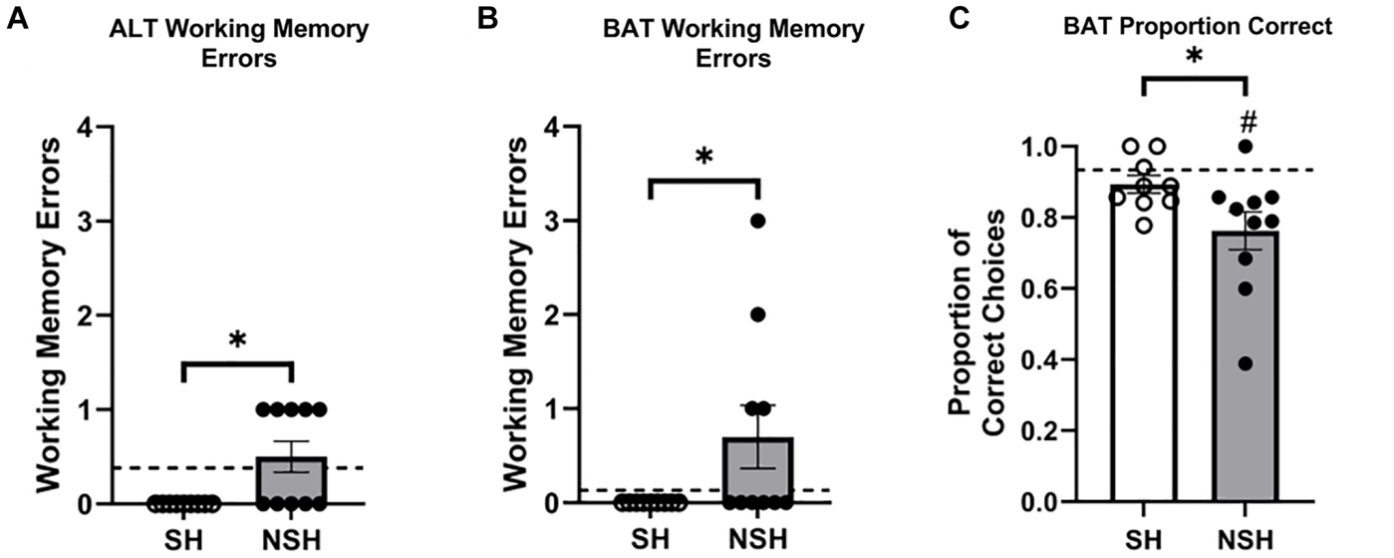
**Performance on test day ALT and BAT.** (**A**) Total number of working memory errors made during the ALT test. (**B**) Total number of working memory errors made during the BAT test. (**C**) Proportion of correct choices made during the BAT test. Abbreviations: ALT: alternation task; BAT: biconditional association task; SH: socially housed; NSH: non-socially housed. Error bars represent standard error of the mean. Dashed lines represent population (test-values) averages obtained from the young cohort and ^#^signifies *p* < 0.05 for these one sample *t*-tests. ^*^signifies *p* < 0.05 for two sample *t*-tests.

One-tailed independent samples *t*-tests revealed significant group differences in the number of working memory errors made both during ALT (t_(17)_ = −2.838, *p* = 0.006, g = −1.245, Figure 3A) and during BAT (t_(9)_ = −2.090, *p* = 0.033, g = −0.867, Figure 3B) on test day. During both tasks, SH rats made significantly fewer working memory errors than NSH rats, mirroring the group differences seen during training and furthering the conclusion that sociality provides benefits for working memory in old age. Additionally, SH rats demonstrated a significantly greater proportion of correct choices made during the BAT test compared to NSH rats (t_(17)_ = 1.934, *p* = 0.035, g = 0.849, Figure 3C), suggesting that long-term social housing protects against the cognitive decline and impaired BAT performance typically observed with advancing age. The order in which the tasks were performed (ALT or BAT test first) did not significantly affect the proportion of correct choices made during the BAT test across groups (main effect of task order: F_(1,15)_ = 1.155, *p* = 0.300, η^2^ = 0.071) or within group (interaction: F_(1,15)_ = 1.341, *p* = 0.265, η^2^ = 0.082), indicating that the order of testing had no differential impact on the results obtained.

### Baseline neuronal activation prior to behavioral testing did not significantly differ across groups

**Figure 4:**
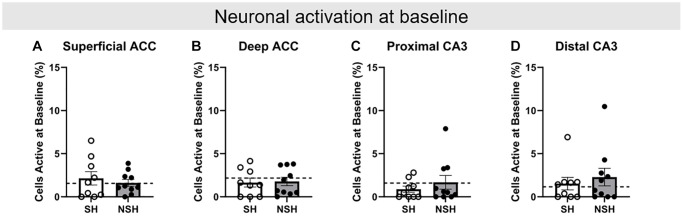
**Baseline neuronal activity prior to task performance on test day.** Percent of neurons active at baseline in the (**A**) superficial ACC, (**B**) deep ACC, (**C**) proximal CA3, and (**D**) distal CA3. Abbreviations: ACC: anterior cingulate cortex; SH: socially housed; NSH: non-socially housed. Error bars represent standard error of the mean. Dashed lines represent population (test-values) averages obtained from the young cohort.

*Arc/Homer*1a double catFISH procedures were used to identify neurons that were active during baseline, ALT, and BAT on test day (Figure 1C). Two-tailed independent samples *t*-tests were conducted to analyze the percent of neurons positive for *Homer*1a mRNA in the cytoplasm in each brain region (ACC superficial, ACC deep, CA3 proximal, and CA3 distal, Figure 1B), indicating activity at baseline while rats were in their home cages. No significant group differences were observed for baseline activity in any brain region (ACC superficial: t_(17)_ = 0.618, *p* = 0.545, g = 0.271; ACC deep: t_(17)_ = −0.184, *p* = 0.856, g = −0.081; CA3 proximal: t_(17)_ = −0.888, *p* = 0.387, g = −0.390; CA3 distal: t_(17)_ = −0.591, *p* = 0.562, g = −0.259, Figure 4), indicating that all groups had similar levels of neuronal activity prior to any behavioral testing and in the absence of cognitive demands additional to those required while the rats are at rest. All further analyses of neuronal activity during behavioral testing are thus normalized to baseline activity.

### Neuronal activation during the alternation (ALT) and biconditional association task (BAT) tests

**Figure 5:**
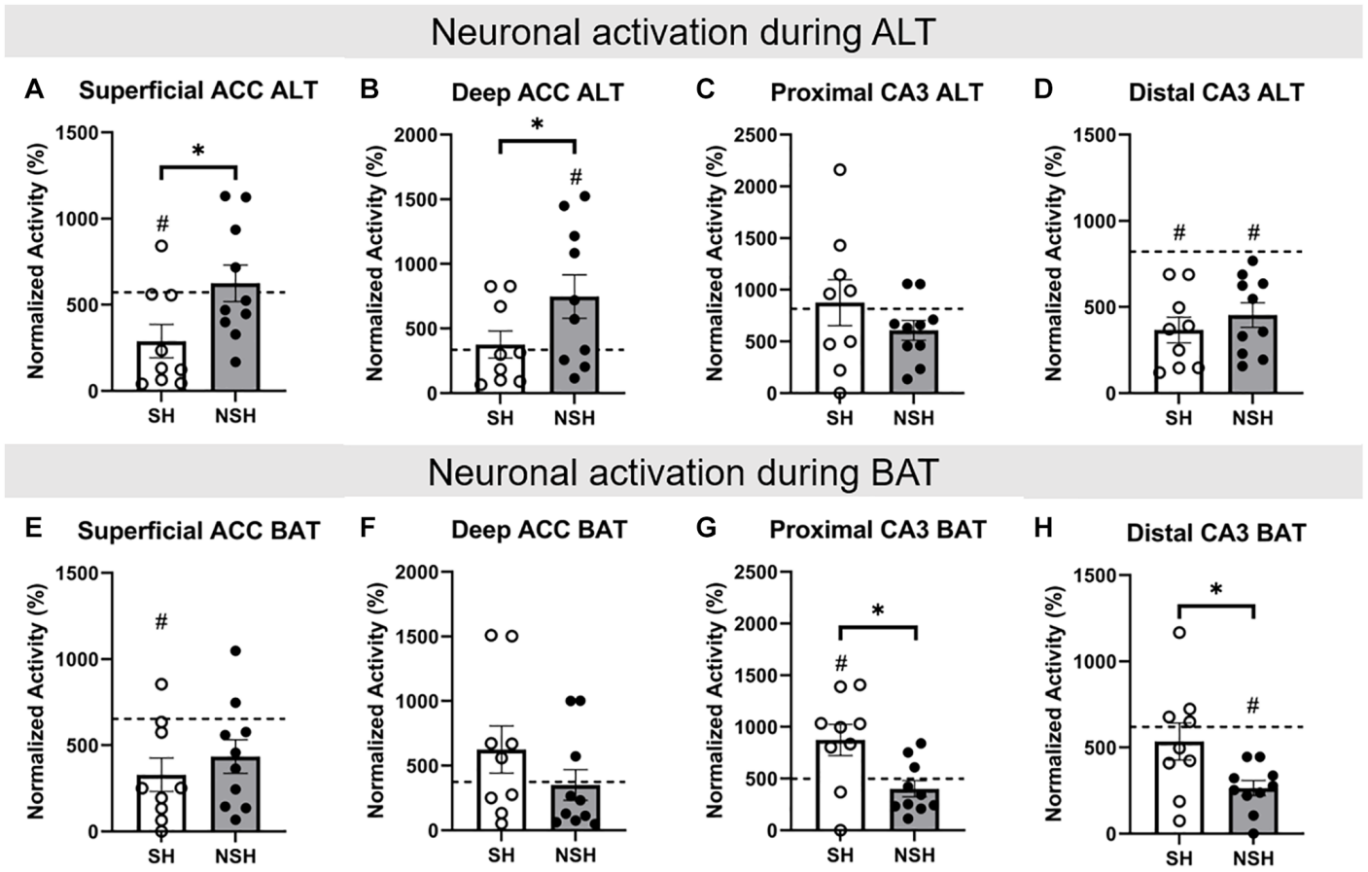
**Percentage of neurons active during ALT and BAT in the ACC and CA3.** All data are normalized to baseline. (**A**) Percent of neurons active in the superficial ACC during ALT. (**B**) Percent of neurons active in the deep ACC during ALT. (**C**) Percent of neurons active in the proximal CA3 during ALT. (**D**) Percent of neurons active in the distal CA3 during ALT. (**E**) Percent of neurons active in the superficial ACC during BAT. (**F**) Percent of neurons active in the deep ACC during BAT. (**G**) Percent of neurons active in the proximal CA3 during BAT. (**H**) Percent of neurons active in the distal CA3 during BAT. Abbreviations: ALT: alternation task; BAT: biconditional association task; SH: socially housed; NSH: non-socially housed; ACC: anterior cingulate cortex. Error bars represent standard error of the mean. Dashed lines represent population (test-values) averages obtained from the young cohort and ^#^signifies *p* < 0.05 for these one sample *t*-tests. ^*^signifies *p* < 0.05 for two sample *t*-tests.

Two-tailed independent samples *t*-tests were performed to assess group differences in the percent of neurons active during the ALT test in the ACC and CA3. Analysis of the percent of cells expressing immediate early genes (IEGs) within the superficial ACC during ALT revealed that SH rats had a significantly lower percent of neurons active during ALT than NSH rats (t_(17)_ = −2.309, *p* = 0.034, g = −1.013, Figure 5A). Analysis of the percent of cells expressing IEGs within deep ACC also revealed that SH rats had a significantly lower percent of neurons active during ALT than NSH rats (t_(14.816)_ = −1.874, *p* = 0.040, g = −0.800, Figure 5B). No significant group differences were found for the percent of neurons active during ALT in the proximal CA3 or in the distal CA3 (t_(17)_ = 1.151, *p* = 0.291, g = 0.505; t_(17)_ = −0.841, *p* = 0.412, g = −0.369, Figure 5C, 5D).

Independent samples *t*-tests were also performed to assess group differences in the percent of neurons active during BAT in the ACC and CA3. No significant group differences were found for the percent of neurons active during BAT in the superficial ACC or in the deep ACC (t_(17)_ = −0.768, *p* = 0.453, g = −0.337; t_(17)_ = 1.288, *p* = 0.215, g = 0.565, Figure 5E, 5F). Analysis of the percent of cells expressing IEGs within the proximal CA3 revealed that SH animals had a significantly greater percent of neurons active during BAT compared to NSH animals (t_(17)_ = 2.855, *p* = 0.011, g = 1.253, Figure 5G). Analysis of the percent of cells expressing IEGs within distal CA3 also revealed that SH animals exhibited a significantly greater percent of neurons active during BAT compared to NSH animals (t_(17)_ = 2.414, *p* = 0.027, g = 1.059, Figure 5H).

**Figure 6:**
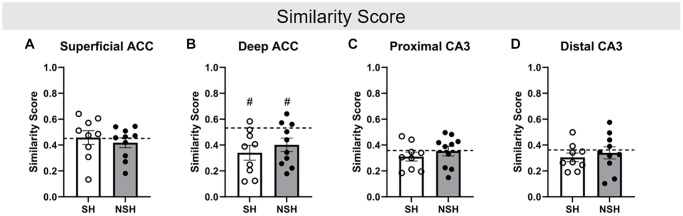
**Similarity score.** Similarity score in the (**A**) superficial ACC, (**B**) deep ACC, (**C**) proximal CA3, and (**D**) distal CA3. Similarity score represents the percent of neurons that were active during both ALT and BAT, while accounting for chance. Abbreviations: SH: socially housed; NSH: non-socially housed; ACC: anterior cingulate cortex. Error bars represent standard error of the mean. Dashed lines represent population (test-values) averages obtained from the young cohort and ^#^signifies *p* < 0.05 for these one sample *t*-tests. ^*^signifies *p* < 0.05 for two sample *t*-tests.

Independent samples *t*-tests were used to examine differences in similarity score, a measure of the percent of neurons active during both ALT and BAT, accounting for chance, within each brain region. No significant group differences in similarity score were observed within any brain region, indicating that social housing did not alter the percent of neurons that were active during both ALT and BAT in these regions of the aged rat brain (ACC superficial: t_(17)_ = 0.780, *p* = 0.446, g = 0.342; ACC deep: t_(17)_ = −0.840, *p* = 0.413, g = −0.369; CA3 proximal: t_(17)_ = −0.791, *p* = 0.440, g = −0.347; CA3 distal: t_(17)_ = −0.487, *p* = 0.633, g = −0.214, Figure 6).

### Aged BAT performance compared to standard young BAT performance

While the primary goal of the current study was to examine differences in performance between aged animals as a factor of long-term social housing, additional analyses were performed to examine whether the performance of our enriched aged animals differed from the standard performance of non-enriched young rats that were 6 months of age at the time of testing. One-sample *t*-tests using the performance of the young rats as the population average revealed that SH animals made significantly fewer working memory errors during BAT training (t_(8)_ = −2.573, *p* = 0.033, d = −0.858, Figure 2C), while NSH animals did not differ from the population average (t_(9)_ = 1.280, *p* = 0.232, d = 0.405, Figure 2C). Interestingly, when the training sessions were divided into early and late trials, SH rats made significantly fewer working memory errors than average during early trials but not during late trials (t_(8)_ = −2.487, *p* = 0.038, d = −0.829; t_(8)_ = 1.639, *p* = 0.140, d = 0.546, Figure 2D), while NSH rats were not significantly different from average during the early trials, but made significantly more working memory errors during late trials (t_(8)_ = 0.238, *p* = 0.818, d = 0.079; t_(8)_ = 2.693, *p* = 0.027, d = 0.898, Figure 2D). The data indicate that while standard young rats show improvements in working memory across a session as they remember the task parameters from the previous session (reference memory), aged NSH rats do not exhibit the normal improvement in working memory across the session. In contrast, social housing seems to improve reference memory, allowing aged SH animals to remember the task parameters of the previous session faster and leading to fewer errors early on in the session. Neither SH nor NSH animals differed from the population average in terms of the proportion of correct choices during BAT training (t_(8)_ = 1.060, *p* = 0.320, d = 0.353; t_(9)_ = −0.408, *p* = 0.693, d = −0.129, Figure 2E) or the number of incorrect trials to criterion (t_(8)_ = 1.888, *p* = 0.096, d = 0.629; t_(8)_ = 2.139, *p* = 0.065, d = 0.713, Figure 2F).

On BAT test day, NSH animals did not differ from the young population average in number of working memory errors made during ALT (t_(9)_ = 0.750, *p* = 0.472, d = 0.237, Figure 3A) or during BAT (t_(9)_ = 1.716, *p* = 0.120, d = 0.543, Figure 3B). Analysis of working memory errors made by SH animals compared to the population was not possible because all SH animals made zero working memory errors during ALT and BAT. Interestingly, NSH rats had a significantly lower proportion of correct choices during the BAT test compared to the population (t_(9)_ = −3.501, *p* = 0.007, d = −1.107, Figure 3C), while the SH rats did not perform differently than average (t_(8)_ = −1.216, *p* = 0.259, d = −0.405, Figure 3C). The order in which the rats performed the ALT and BAT tests did not significantly influence the proportion of correct choices made during the BAT test across groups (main effect of task order: F_(1,21)_ = 0.001, *p* = 0.970, η^2^ = 0.000) or within group (interaction: F_(2,21)_ = 2.631, *p* = 0.096, η^2^ = 0.200), indicating that the order of testing had no differential impact on the results obtained.

### Aged neuronal activation compared to standard young neuronal activation during the alternation (ALT) and biconditional association task (BAT) tests

Analyses were performed to examine how the percent of neurons active in the aged rats during the behavioral tests (ALT and BAT) compare to the standard percent of neurons active in the non-enriched young adult rats, again using one-sample *t*-tests with the population average generated from the young rats. Importantly, no significant differences in baseline neuronal activation in any brain region for either SH or NSH rats were found compared to the population average (SH: superficial ACC t_(8)_ = 0.761, *p* = 0.468, d = 0.254; deep ACC t_(8)_ = −1.013, *p* = 0.341, d = −0.338; proximal CA3 t_(8)_ = −2.007, *p* = 0.080, d = −0.669; distal CA3 t_(8)_ = 0.517, *p* = 0.619, d = 0.172; NSH: superficial ACC t_(9)_ = 0.173, *p* = 0.867, d = 0.055; deep ACC t_(9)_ = −0.801, *p* = 0.444, d = −0.253; proximal CA3 t_(9)_ = 0.115, *p* = 0.911, d = 0.036; distal CA3 t_(9)_ = 1.106, *p* = 0.297, d = 0.350, Figure 4).

When comparing the percent of neurons active during the ALT test, SH animals demonstrated a significantly lower percent of cells active in the superficial ACC compared to the population average (t_(8)_ = −2.926, *p* = 0.019, d = −0.975, Figure 5A), whereas NSH animals did not show significantly different activation from the population (t_(9)_ = 0.487, *p* = 0.638, d = 0.154, Figure 5A). Within the deep ACC, NSH animals had a significantly greater percent of cells active compared to the population (t_(9)_ = 2.446, *p* = 0.037, d = 0.773, Figure 5B), but SH animals did not (t_(8)_ = 0.384, *p* = 0.711, d = 0.128, Figure 5B). Together these data suggest that social housing may prevent the aged brain from overactivity in the ACC as is seen in the NSH rats. Neither SH nor NSH animals had a significantly different percent of neurons active in the proximal CA3 during ALT compared to the population (t_(8)_ = 0.275, *p* = 0.790, d = 0.092; t_(9)_ = −2.162, *p* = 0.059, d = −0.684, Figure 5C). Both SH and NSH animals displayed a lower percent of neurons active in the distal CA3 during ALT compared to the population average (t_(8)_ = −6.125, *p* < 0.001, d = −2.042; t_(9)_ = −5.181, *p* < 0.001, d = −1.638, Figure 5D). This finding mirrors the hypoactivity of distal CA3 neurons previously seen in aged rats [26] but suggests that social housing does not affect this decreased activity in old age. Therefore, increased activity in the deep ACC in NSH rats rather than altered activity in the CA3 may be linked to impaired working memory during the ALT task.

When comparing the percent of neurons active during the BAT test, SH animals had a significantly lower percent of cells active in the superficial ACC compared to the population average (t_(8)_ = −3.344, *p* = 0.010, d = −1.115, Figure 5E), whereas NSH animals did not (t_(9)_ = −2.231, *p* = 0.053, d = −0.645, Figure 5E). Neither SH nor NSH animals had a significantly different percent of neurons active in the deep ACC during BAT compared to the population (t_(8)_ = 1.373, *p* = 0.207, d = 0.458; t_(9)_ = −0.207, *p* = 0.841, d = −0.065, Figure 5F). Within the proximal CA3, only SH rats had a significantly greater percent of neurons active during BAT compared to the population (t_(8)_ = 2.483, *p* = 0.038, d = 0.828, Figure 5G), whereas the NSH rats did not (t_(9)_ = −1.230, *p* = 0.250, d = −0.389, Figure 5G). Within the distal CA3, only NSH animals had a significantly lower percent of neurons active during BAT (t_(9)_ = −8.113, *p* < 0.001, d = −2.566, Figure 5H), while SH animals did not (t_(8)_ = −0.799, *p* = 0.447, d = −0.266, Figure 5H). These results indicate that aging without sociality contributes to a decrease in the number of distal CA3 neurons active during BAT while social housing prevents this decrease and further increases the number of proximal CA3 neurons active during BAT in old age. Fewer neurons active in the CA3 in the NSH aged rats may therefore underlie their impaired performance on the BAT test while increased CA3 activity likely protects the SH aged rats from these impairments.

Finally, analyses of similarity score revealed that both SH and NSH animals had a significantly lower similarity score for neurons in the deep ACC compared to the population (SH: t_(8)_ = −3.243, *p* = 0.012, d = −1.081; NSH: t_(9)_ = −2.522, *p* = 0.033, d = −0.797, Figure 6B), indicating that the aged animals had fewer of the same deep ACC neurons active during both ALT and BAT compared to the young animals. This finding is consistent with previous research demonstrating that aged rats have lower similarity scores compared to young rats and suggests that young rats may be able to recruit a similar population of deep ACC neurons to perform distinct but related behaviors while aged animals may be impaired in this ability [31]. However, no significant differences in similarity score were found for the superficial ACC (SH: t_(8)_ = 0.279, *p* = 0.788, d = 0.093; NSH: t_(9)_ = −0.877, *p* = 0.403, d = −0.277, Figure 6A), proximal CA3 (SH: t_(8)_ = −1.343, *p* = 0.216, d = −0.448; NSH: t_(9)_ = −0.130, *p* = 0.899, d = −0.041, Figure 6C), or distal CA3 (SH: t_(8)_ = −1.681, *p* = 0.131, d = −0.560; NSH: t_(9)_ = −0.558, *p* = 0.591, d = −0.176, Figure 6D). This lack of difference in similarity score between the young and aged animals in a subregion specific manner may be the result of all aged animals having received EE. Previous research has demonstrated that EE has positive effects on cognition in old age [39, 40, 42, 45, 49–51, 53, 54]. Therefore, the EE received by the aged animals but not the young animals may have largely preserved the ability of the aged animals to recruit a similar population of neurons for both ALT and BAT, like the young animals. Additionally, since similarity scores were not different between the SH and NSH rats, EE may play a greater role in preserving the recruitment of the same neurons for related tasks than social enrichment.

## DISCUSSION

This study was designed to investigate the effect of long-term social housing on cognitive decline and neuron ensemble activity dynamics in old age. All aged rats received EE but were either socially housed or non-socially housed for the entirety of their lives. All subjects were trained and tested on ALT and BAT and double catFISH was utilized to allow for the analysis of the percent of neurons active during behavioral testing. SH rats made fewer working memory errors during BAT training and during ALT and BAT testing than NSH rats, demonstrating that social housing protects against working memory deficits that are widely reported to occur in aged rats [2, 20, 46, 62, 65, 67]. Additionally, SH rats had a higher proportion of correct choices compared to NSH rats during the BAT test, and while the NSH rats had significantly impaired performance on BAT compared to the standard young rats as has been reported previously [31, 32], SH rats did not show this same impairment indicating that social housing may also provide benefits for cognitive flexibility in old age above and beyond the benefits provided by EE alone.

### Social housing protects against decline in working memory in old age

Working memory declines with advancing age across species [2]. Here, we found that aged NSH rats made significantly more working memory errors than aged SH rats during BAT training, specifically during the later trials within a session when rats rely more fully on working memory rather than long-term reference memory. Interestingly, during the late trials, NSH rats also showed significant impairments in working memory when compared to the population average of young individually housed rats, while aged SH rats were not different from the population standard. These findings align with previous research indicating that working memory declines with advanced age [1, 2, 4], while also demonstrating that long-term social housing mitigates this decline. They are also further consistent with results showing that memory systems adapt dynamically over the course of a trial to minimize cognitive effort [85]. A previous experiment utilizing these same subjects also revealed that SH rats committed fewer working memory errors in old age than NSH rats on a radial arm maze task, but there were no differences in reference memory errors between the groups in old age [46]. In the current study, SH rats also made significantly fewer working memory errors than NSH rats during both the test day ALT and the BAT tests. Together these data support the conclusion that sociality protects against the decline in working memory in advanced age.

### Social housing preserves biconditional association task (BAT) performance in old age

Not only does sociality confer working memory benefits in advanced age, but long-term social housing also appears to provide benefits to complex cognition. BAT is a cognitively taxing task that requires rats to associate the correct object with its location in the maze. Rats must be cognitively flexible to switch between responding to one correct object in the left arm of the maze and a different correct object in the right arm of the maze. It has been established that aged rats are significantly impaired on this task because their ability to make these complex, condition-dependent associations deteriorates with advancing age [32]. In line with our hypothesis, the results of the current study showed that SH rats performed better on the BAT test than NSH rats, as indicated by a significantly higher proportion of correct choices during the test. Interestingly, when compared to the population average, NSH rats were significantly impaired on this task- replicating previous findings [32]- however, the SH rats did not show impairments compared to the population. These findings suggest that long-term social housing, rather than EE alone, preserves cognitive flexibility in old age. The fact that group differences between SH and NSH rats were not observed during BAT training could potentially be due to a low proportion of correct choices for all groups while learning the task parameters. Significant differences during the BAT test session may therefore more accurately reflect differences in cognitive flexibility since all subjects successfully acquired the task prior to the test session. It is also important to note that the EE received by all aged rats, even in the absence of long-term social housing, may have had some protective effects and allowed both SH and NSH animals to effectively learn BAT and perform similarly to the young animals during training. These findings provide evidence that, although the ability to perform cognitively complex tasks typically declines with advancing age, social housing protects against the loss of cognitive flexibility above and beyond EE without sociality.

### Social housing affects neuronal activity in the ACC and CA3 during the alternation (ALT) and biconditional association task (BAT) tests, respectively

The differences in working memory and BAT performance between SH and NSH rats are reflected in group differences in the number of neurons active during the ALT and BAT tests. Specifically, during the test day ALT task, SH rats exhibited a significantly lower percent of neurons active compared to NSH rats in both the superficial and deep layers of the ACC, but not in the CA3. Additionally, SH rats had a significantly lower percent of neurons active in the superficial ACC during the ALT test than the population average, while NSH rats had a significantly greater percent of neurons active in the deep ACC than the population average. Previous work has implicated the cingulate cortex in working memory in both humans and rodents [86–90]. While some studies have found less activity in individuals with poorer working memory [87], as well as decreased ACC activity in old age [91], our findings suggest that fewer ACC neurons active during the ALT working memory task may conserve working memory in old age in the SH rats. This finding can be interpreted in light of the neural efficiency hypothesis which suggests that individuals may need to allocate differing amounts of mental resources to perform a cognitive task depending on innate capabilities, so neural activation may be more strongly related to the mental workload required to perform a task rather than task performance itself [92]. Interestingly, while the difference in working memory errors during the ALT test between the SH and NSH rats was significant, NSH rats still performed well on this task, with all NSH subjects making either zero or one working memory errors during this test, again potentially resulting from a somewhat protective effect of EE even in the absence of sociality. Therefore, the greater percent of ACC neurons activated during this working memory task may represent a greater mental workload in the NSH rats compared to the SH rats and indicate that long term social housing leads to more efficient neuronal activation to accommodate relatively preserved cognitive task performance in old age. Interestingly, the reduced activity in the SH rats was observed in both subregions of the ACC, indicating that the effect of sociality on ACC neuronal activity during ALT is not subregion dependent.

It is important to note that the current study used labeling of two immediate early genes as an indication of which neurons were active during ALT and BAT. While this method allows for the identification of how many distinct neurons were activated during each task, it does not provide information as to whether the same neuron was active multiple times within one task or if more neurons were recruited during correct vs. incorrect trials. It is therefore possible that SH rats required greater activity from a fewer number of neurons to successfully perform ALT while NSH rats had to recruit a larger number of different neurons to perform this simple working memory task. Future studies should parse out the difference between the percent of distinct neurons that were active within each task and the overall neuronal activation during the task using methods such as *in vivo* calcium imaging. Interestingly, we found that both NSH and SH aged rats used fewer of the same neurons in the deep ACC for ALT and BAT compared to the young animals, as demonstrated by a lower similarity score. This suggests a higher neural efficiency for the young rats, as they are able to rely on the same deep ACC neurons for multiple tasks.

During the BAT test, SH rats had a significantly greater percent of neurons active compared to NSH rats in both the proximal and distal regions of the CA3, but not in the ACC. Additionally, SH rats had a significantly greater percent of neurons active in the proximal CA3 during the BAT test than the population average, while NSH rats had a significantly lower percent of neurons active in the distal CA3 than the population average. Although age-related increases in neuronal activity have been reported for CA3 [11, 15, 23, 24], a recent neurophysiology study showed that age-related changes in neuronal activity in the CA3 may be subregion dependent, where aged rats have higher firing rates in the proximal CA3 but lower firing rates in the distal CA3 compared to young animals [26]. The authors suggest that in these aged rats, the proximal CA3 may shift from its role in pattern segregation to pattern completion. Interestingly, in the current study, NSH rats show a decrease in the proportion of distal CA3 neurons active during BAT compared to young rats, but SH do not. This suggests that long-term social housing may preserve the pattern of neuronal activity in the distal CA3, allowing the distal CA3 to maintain its role in pattern completion and preventing the proximal CA3 from shifting away from pattern segregation. Pattern segregation is fundamentally associated with BAT performance as rats need to distinguish between similar but distinct contexts to choose the correct object. Therefore, intact pattern segregation would allow the SH animals to successfully differentiate between their memories to determine which object is correct in their current location in the maze. While pattern completion is also important for BAT performance and allows animals to retrieve a complete memory of the correct object choice based on their current location in the maze from partial cues, overreliance on pattern completion with a lack of pattern segregation may cause overgeneralization and confusion between the similar contexts and may lead to difficulty associating the correct choice with the context. As distal CA3 neurons show reduced activity in NSH animals, proximal CA3 neurons may shift away from pattern segregation to play a greater role in pattern completion, and NSH animals may overgeneralize and therefore have trouble distinguishing between correct objects in this cognitively complex task. While there were clear differences in CA3 neuronal activity between the SH and NSH animals during BAT, no differences were detected in the percent of neurons active in the ACC during BAT. This may be due to limitations in the methods of the current study, as BAT measures only one aspect of complex cognition. Indeed, another study investigating the effect of aging on ACC neuronal activity using the methods of BAT and catFISH was similarly unable to detect differences in the percent of active neurons between young and aged rats [19], although all of these rats were single housed in standard (non-EE) conditions. Therefore, future studies should also investigate whether sociality provides benefits for additional aspects of complex cognition in advanced age and whether the ACC and CA3 are essential for modulating those effects.

### Limitations and future directions

One limitation of the current study is that only male rats were used. Future studies should investigate the effects of long-term social housing in addition to EE on cognitive decline with advancing age in females to further generalize our findings. Additionally, while the current investigation into the neurobiological mechanisms underlying the protective effect of social housing on memory in old age focused on the ACC and CA3, previous studies have also shown that other regions in the PFC and MTL, including the medial PFC and CA1 [31], are important for BAT, and future studies should examine whether social housing may attenuate age-related cognitive decline through altered neuronal activity in these regions as well. Another limitation is the lack of an enriched group of young rats. While a group of young, individually housed rats with no EE was included to represent the population average of rats which are typically used in aging neuroscience research, both groups of aged rats received EE in their home cages and performed an array of cognitive testing prior to the experiments described here (for details on prior cognitive testing, see [41, 46, 52, 69]). As previous studies have identified the importance of EE for cognition [36, 38–40, 42, 45, 46], the presence of EE in conjunction with the performance of cognitive and behavioral experiments may have increased the number of superagers in each of the SH and NSH groups. Therefore, it is somewhat difficult to determine whether differences in cognitive performance and neuronal activation between the aged groups and the young population average are due to aging or environmental factors. However, since the primary focus of this study was to isolate the effect of the sociality aspect of enrichment and investigate its effects on cognition with advanced age, this limitation does not take away from the finding that aged SH rats demonstrated a significant improvement in working memory and cognitive flexibility compared to the aged NSH rats. Therefore, despite the lack of an enriched group of young rats, the current study expands our understanding of the benefits of social enrichment in the context of aging and provides compelling evidence for the importance of sociality independent from other aspects of enrichment for preserving cognition in advanced age. Future studies should further investigate how aged animals receiving EE and long-term social housing compare to young animals also receiving EE and social housing.

To our knowledge, this is the first study to investigate whether the ACC and CA3 modulate the protective effects of sociality on age-related cognitive decline in rats using BAT. Because the PFC and hippocampus are some of the first brain regions to show age-related cognitive decline [8–10], aged rats are typically impaired on BAT [29–32], and sociality is known to protect against cognitive decline with age [69–71], we hypothesized that the ACC and CA3 may modulate the beneficial effect of long-term social housing on cognition in advanced age. The current results indicate that social interaction throughout the course of one’s life may attenuate the normal cognitive decline in old age above and beyond EE alone. Despite receiving EE, NSH rats made more working memory errors and had a lower proportion of correct choices on BAT than the SH rats, indicating that social housing likely provides benefits to working memory and cognition in advanced age. Further, SH animals had a significantly lower percent of neurons active in both the superficial and deep layers of the ACC compared to the NSH animals during the ALT test, and a significantly greater percent of neurons active in both the proximal and distal CA3 during the BAT test, providing insight into some potential neurological mechanisms by which long term social housing attenuates cognitive decline during normal aging. Understanding the contributions of proximal and distal layers of the CA3 and of deep and superficial layers of the ACC can provide insights into the neural mechanisms underlying cognitive flexibility in advanced age, emphasizing the need for further research in this area.

## MATERIALS AND METHODS

### Subjects and housing

20 male Long-Evans rats *(Rattus norvegicus)* arrived from Charles River Laboratories on post-natal day (PND) 21. The supplier reported with reasonably high certainty that all animals came from separate litters. All rats had the base of their tails marked with colored marker to assist with identification. Upon arrival, the rats were immediately randomly assigned to either the socially housed (SH) or non-socially housed (NSH) group. One SH rat died of natural causes (PND 264) leaving nine rats in the SH group and 10 rats in the NSH group. One NSH rat often tried to escape the maze and would freeze and defecate, producing few data points, so his data were removed in cases where his results were outliers (+/-SD above or below the mean); this occurred for analyses of working memory errors during early versus late trials and incorrect trials to criterion (see Data Analysis for outlier analysis) but his data were included in all other behavioral and neural analyses. Rats were housed for approximately two weeks in plastic shoebox cages, NSH rats individually, and SH rats in two groups of five. On PND 35, when rats were large enough to not escape the wired enriched caging, rats were housed permanently in enriched housing. All SH rats were housed together in a cage (36 × 24 × 63 inches), and each NSH rat was housed individually in a slightly smaller but otherwise identical cage (17 × 12.75 × 24 inches) (Ferret Nation). SH rats had continual access to their cage mates, except when individuals were temporarily taken out of the home cage for behavioral testing. NSH rats never had direct access to other rats, however, the housing conditions allowed them to smell, hear, and see other rats to avoid complete social isolation and better represent human conditions. Both SH and NSH rats had constant access to enrichment in their cages. The large cages enclosed by metal bars with multiple leveled platforms provided ample opportunities for climbing and exercise for both the SH and NSH rats. The cages included running wheels, plastic toys and enclosures, an open-topped plastic shoebox cage with Corncob bedding (17 × 8 × 8 inches) and wooden chew toys. Corncob bedding was provided at the bottom of all plastic enclosures (Enrich-o’cobs) and at the bottom of the wire caging (although because of the wire floor, rats didn’t have physical contact with this bedding). All subjects were housed in one colony room maintained at 21.6 degrees Celsius and kept on a 12:12 reversed light:dark cycle, with light onset at 8 pm and light offset at 8 am. Subjects had constant access to water ad libitum. Rat chow (LabDiet) was provided ad libitum until rats reached adulthood (approximately PND 60), at which point weighed rations of chow were provided daily to approximate 90% of free-feeding weight. Food was provided in several different locations in the large social cage and the individual cages for all rats to forage. If a social rat’s weight was significantly below the average or decreased over time, that rat was taken out of the cage during feeding times and given an hour to eat alone in a shoebox cage before being placed back into the social cage. Rats were weighed weekly and chow rations were provided after testing each day.

SH and NSH subjects also received cognitive enrichment as they were not experimentally naïve. All subjects were part of a larger study and performed identical, unrelated behavioral tests prior to the testing described in this article. These experiments included various memory and spatial navigation tasks such as the social interaction paradigm performed from PND 391–393 [69], the radial arm maze performed from PND 308–325, 475–493, and 699–717 [46], the continuous T maze performed from PND 654–658 [52], and the Barnes maze performed from PND 142–168, 427–513, and 671–722 [41]. Briefly and respectively, SH subjects showed significantly less preference for social interactions, made significantly fewer working memory errors in old age, were less apt to maladaptively perseverate, and showed increased acquisition in adulthood than their NSH counterparts.

An additional eight male Long-Evans rats arrived from Charles River Laboratories on PND 21, 19 months after the arrival of the original 20 rats. These rats were housed individually in a standard plastic shoebox cage (17 × 8 × 8 inches) and topper with a tunnel toy and Corncob bedding. This additional young group performed BAT in adulthood on PND 183 (6 months), as many studies conduct behavioral testing on adult rats that are individually housed with little enrichment. The group of young rats was added so that the performance and neuronal activation of aged rats (26 months) could be compared to standard young performance and neuronal activation. All procedures described in the current study were approved by the Institutional Animal Care and Use Committee (IACUC) of Providence College under protocol VLT090314.

### Biconditional association task (BAT)

All training and testing sessions occurred inside a two-arm Y-shaped maze (Figure 1A). The walls of the maze were constructed out of MegaBlok Legos and the maze was placed on a wooden platform sealed with waterproof paint. The base of the maze measured 40 × 20 cm, each arm measured 85 × 8 cm, and the chambers at the end of each arm measured 35 × 25 cm. Two food wells, each one cm deep, were drilled into the base of the maze in each chamber. Wells served as reward containers and allowed objects to be placed over the wells during discrimination and BAT testing. Froot loops were crushed and distributed in each food well and whole fruit loops were taped under the board to prevent rats from discriminating based solely on the scent of the Froot loop reward. Each arm had two distinct allocentric visual cues to allow subjects to discriminate between arms; the left arm was built with blue and green Megablocks in a striped pattern and featured two blue zig zag marks on the floor, while the right arm was built with red and yellow Megablocks in a sporadic pattern and featured two red vertical lines on the floor. The arms of the maze were separated by a removable poster board to prevent the rat’s vision of the researcher baiting the opposite arm. Objects were cleaned with 70% ethanol between trials and different versions of all objects were used so that alternations were continuously presented.

#### Alternation (ALT) training

Rats were first trained to alternate between the right and left arms of the maze. The subject was randomly placed in one arm of the maze while the opposite arm was baited with half of a Froot loop as a reward. Once the rat alternated arms and received the food reward, the opposite arm was baited. Since there were two food wells in each arm, food rewards were randomly placed in either the left or right food well. No objects were present during ALT training, and rats could freely travel between the arms of the maze. The rats reached criterion when they could successfully alternate arms of the maze 32 times within 30 minutes. When a rat failed to alternate and re-entered the previously visited arm, the error was recorded as a working memory error.

#### Object control/discrimination training

Rats were next trained to discriminate between objects and displace the correct object to receive a food reward. Two different objects, A and B, were placed covering the food wells in the choice platforms at the end of each arm of the maze. For half of the rats, object A was the rewarded stimulus, and for the other half object B was the rewarded stimulus. At the start of the training session, the rat was randomly placed in one arm while objects were placed in the choice platform of the other. All rats had to learn to alternate arms and displace the correct object to expose the food well with the reward. If the rat displaced the correct object, the choice was recorded as correct, and the rat received the food reward. If the rat displaced the incorrect object, the choice was recorded as incorrect, and the rat did not receive a reward. If the rat did not displace any objects, the response was recorded as no choice and the rat did not receive a reward. If the rat entered the choice platform of the incorrect arm, the response was recorded as a working memory error. After an object was displaced, the objects and any remaining rewards were removed from the arm to prevent multiple responses. A reward and the objects were then placed in the alternate arm for the next trial. The next trial began when the rat entered the starting platform at the beginning of the arm and then traveled down the arm to the choice platform. Criterion was reached when the rat successfully alternated arms and displaced the correct objects 26 out of 32 trials in a single session.

#### Biconditional association task (BAT) training

Rats were next trained on BAT. Two new objects, C and D, were used. BAT training was identical to discrimination training except that the conditions for the correct object differed; for BAT the correct object depends on its location within the maze and requires the rat to associate the object with the location. For example, object C may be the correct choice in the left arm of the maze, but object D would be the correct choice in the right arm of the maze. Again, the placement of the objects over the left and right food well was randomized and counterbalanced for each trial. Criterion was reached when rats made ≥13 correct choices in the left arm and ≥13 correct choices in the right arm out of 32 total trials on two consecutive days.

#### Retention task

48 hours after the rats reached criterion for BAT training, a retention task was administered to ensure criterion performance was maintained for test day. For the retention task, rats performed a repeat of BAT and were required to reach a criterion of 26 correct trials in one day regardless of the number of total trials required. Since not all of the rats reached criterion for BAT training at the same time, rats that reached criterion for BAT training earlier received additional retention sessions to ensure all rats were proficient at the task on test day. Retention tasks were administered every third day until 24 hours immediately prior to the final test day.

#### Test

The final test was administered 24 hours after the last retention task. The test consisted of five minutes of epoch one, a twenty-minute inter-epoch interval, and then five minutes of epoch two. All rats completed one epoch of ALT and one epoch of BAT, with the order of tasks counterbalanced across animals. All rats were tested in a global yolk fashion where the first rat performed the tasks for five minutes and all following rats performed approximately the same number of trials. Specifically, because the first aged rat completed ten trials of each task in five minutes, all subsequent rats received ten trials for each task. Immediately following the completion of the second epoch of the test, each rat was taken into the surgical wet lab to be euthanized. Strict testing times were planned and adhered to maximize the number of brain extractions that could occur in one day. Testing and brain extractions occurred over two days. All SH rats were tested on the first day to avoid possible stress to the remaining SH rats if half of the rats were removed from the cage. All NSH rats were tested on the second day, and half of the young rats were tested on each day.

### *Arc/Homer*1a compartmental analysis of temporal activation with fluorescence *in situ* hybridization (catFISH)

Neurobiological data were collected using *Arc/Homer*1a double catFISH to identify neuron activity-dependent expression of immediate-early genes (IEGs). Euthanasia and brain extraction occurred immediately after the conclusion of the final test (second epoch). Rats were placed in a bell jar with isoflurane-saturated cotton. The rats were separated from the isoflurane-saturated cotton by a wire mesh shield. Once the rats lost the righting reflex, they were euthanized by rapid decapitation using a guillotine. Brains were extracted and flash frozen in 2-methyl butane chilled in a bath of dry ice with 100% ethanol (−70°C). The tissue was stored at −80°C until it was sliced by a cryostat at 20-μm thickness. The slices were thaw-mounted on Superfrost Plus Slides.

Tissue was processed for fluorescence *in situ* hybridization to identify *Arc* and *Homer*1a mRNA. A commercialized *in vitro* transcription and labeling kit was used to synthesize two riboprobes, one digoxigenin-labeled riboprobe for *Arc* and one fluorescein-labeled riboprobe for *Homer*1a. Riboprobes were generated using linearized and PCIA purified plasmid *Arc* and *Homer*1a cDNA. A RNase free technique, nuclease free reagents, UV-treated RNase free water, and nuclease free equipment were used throughout the duration of the procedure. The riboprobes were added to the brain tissue and the tissue was incubated for approximately 20 hours. Anti-digoxigenin was added to the tissue to detect *Arc* mRNA and anti-fluorescein was added to detect *Homer*1a mRNA. Nuclei of the neurons were counterstained with DAPI.

A Keyence fluorescence microscope BZ-X series was used to take z-stacks of the brain tissue at increments of 1μm with a 40x objective lens. The right hemisphere of the brain was imaged for each rat. Four brain regions were examined for all rats: superficial and deep layers of the ACC, and proximal and distal regions of the CA3 region of the hippocampus (Figure 1B). Approximately three images were collected per region for each rat (Table 1). Images of the ACC were taken around 0.24 mm anterior to bregma with all images collected between 2.52 mm anterior to bregma and 0.60 mm posterior to bregma. Images of the CA3 were taken around 3.12 mm posterior to bregma with all images collected between 2.52 mm and 4.20 mm posterior to bregma.

**Table 1:** Total number of images analyzed and average number of cells counted for each region.

	**Superficial ACC**	**Deep ACC**	**Proximal CA3**	**Distal CA3**
**Number of images, total**				
Young	24	24	24	24
Socially housed	25	27	25	25
Non-socially housed	29	30	30	28
**Average number of cells analyzed**				
Young	250.75	218.63	237.75	293.25
Socially housed	224.67	203.11	185.67	271.67
Non-socially housed	245.00	213.30	242.80	253.10
One-way ANOVA (F; *p*)	0.390; 0.682	0.112; 0.895	3.362; 0.052	0.876; 0.429

One-way ANOVAs revealed no significant differences in the number of cells counted for each group within any of the brain regions assessed.

*Arc* and *Homer*1a mRNA were quantified as described previously [31]. Briefly, each image was manually quantified using ImageJ Software and by experimenters blind to subject group to identify the total number of neurons and the neurons positive for *Arc* and *Homer*1a mRNA. Experimenters were trained on cell counting procedures prior to the quantification of experimental images to ensure inter-rater reliability. To obtain the total number of neurons, nuclei stained with DAPI were counted with the *Arc* and *Homer*1a channels off. Once the total number of neurons was determined, the *Arc* and *Homer*1a channels were turned on and cells were classified as positive for *Arc* and *Homer*1a in the nucleus, cytoplasm, or both. These IEGs were counted as present in the nucleus if 1 or 2 fluorescently labeled foci were detected within the nucleus on 4 or more consecutive planes. IEGs were counted as present in the cytoplasm if fluorescent labeling above background was detected surrounding at least 1/3 of the nucleus on 2 or more consecutive planes (Figure 1D). The transcription kinetics of *Arc* and *Homer*1a are distinct, such that they can be used to infer the neuronal activity patterns of large ensembles of neurons at two distinct 5 min epochs of behavior separated by 20–30 min. *Arc* transcription foci can be identified in the nucleus of the neuron 1–2 minutes after patterned neuronal firing that is associated with active behavior. 20–30 minutes following neuronal activity, *Arc* mRNA translocates to the cytoplasm where it can be identified as a halo around the nucleus. *Homer*1a has transcription kinetics that are distinct from *Arc* such that the foci are not evident until approximately 30 minutes after cellular activity, which corresponds to the time point at which *Arc* mRNA in the cytoplasm are evident. *Homer*1a also translocates to the cytoplasm, approximately 60 minutes after neuronal activity [72, 75]. Therefore, the subcellular location of the immediate early genes in the neurons indicate during which epoch of behavior (ALT or BAT) the neuron was active [72–74]. If a cell was positive for *Homer* 1A in the cytoplasm, the neuron was active at baseline 60 minutes before euthanasia; if the cell is positive for *Arc* in the cytoplasm and *Homer*1a in the nucleus, the neuron was active during epoch one 30 minutes before euthanasia; if a cell is positive for *Arc* in the nucleus, the neuron was active during epoch two right before euthanasia; if a cell is positive for *Arc* and *Homer*1a in the nucleus and *Arc* in the cytoplasm, the neuron was active during both epochs one and two; if a cell is positive for *Arc* and *Homer*1a in the nucleus and *Arc* and *Homer*1a in the cytoplasm, the neuron was active during both epochs one and two and at baseline (Figure 1C). The number of neurons active during each behavioral task was averaged across the three images collected for each brain region to determine the percent of neurons active during each behavioral task for each rat.

### Data analysis

Three behavioral measures during BAT were analyzed: number of working memory errors (i.e., re-entries into the previously baited arm), proportion of correct choices for each task, and number of incorrect trials until criterion was reached. The *a priori* hypothesis was designed to examine the effect of long-term social housing on cognitive decline in old age and proposed that aged SH rats would show less cognitive decline than NSH rats. One-tailed independent samples *t*-tests between the aged SH and aged NSH rats were conducted to test this hypothesis.

For analysis of neurons active during ALT and BAT, an average percent of active neurons in each brain region (ACC superficial, ACC deep, CA3 proximal, and CA3 distal) was calculated for each rat. ALT activity refers to neurons that were active only during ALT while BAT activity refers to neurons that were active only during BAT. For each rat, neuronal activity during ALT and BAT was normalized to baseline neuronal activity to control for neurons that were active in the absence of a cognitive task. Baseline activity refers to the percent of neurons active in the home cage before any cognitive tasks were administered. Normalized activity for each rat was calculated by dividing the neuronal activity during a task (ALT or BAT) by the average group neuronal activity at baseline. Neuronal activity for each rat was divided by the average baseline activity of the group rather than baseline activity of the individual because some subjects had zero activity at baseline. Two-tailed independent samples *t*-tests were performed to assess differences in neuronal activation in each brain region of interest during ALT and BAT as a factor of long-term social housing. Similarity scores were also calculated to examine differences in the percent of neurons active during both ALT and BAT, accounting for chance. Similarity scores were calculated as:


$$\frac{pB-(pBAT\times pALT)}{\frac{pBAT+pALT}{2}-(pBAT\times pALT)}$$

where pBAT, pALT, and pB are the proportions of neurons active during BAT, ALT, and both tasks, respectively. Two-tailed independent samples *t*-tests were performed to assess differences in similarity score between SH and NSH rats.

While the primary aim of the present study was to investigate the effect of social housing on behavioral and neurobiological changes in aged rats, an additional group of young, non-socially housed rats was included to allow for the comparison of behavior and neuronal activation of aged rats to that of standard young adult rats. Therefore, additional one-sample *t*-tests using young rat behavioral performance and neuronal activity as population averages were performed to compare the SH and NSH aged animals with field standards.

All statistics were conducted using the Statistical Package for Social Sciences (SPSS). Proportions were arcsine transformed before statistical analysis to better approximate the normality assumption underlying parametric statistics [93]. Outliers were identified as any data point more than two standard deviations above or below the mean and were replaced with a value one unit greater or less than the value of the next largest or smallest data point within two standard deviations of the mean [94, 95]. The assumption of homogeneity of variance was tested for all statistical comparisons and was corrected for when the assumption of homogeneity of variance was violated. Effect sizes are reported as Cohen’s d for one sample *t*-tests and for independent samples *t*-tests where sample sizes are equal and as Hedges’ g for independent samples *t*-tests where sample sizes are unequal. All analyses used *p* < 0.05 to signify statistical significance. Based on our previous studies with these aged SH and NSH rats [41, 46, 52, 69] and our corresponding hypothesis that SH rats would outperform NSH housed rats behaviorally, all behavioral *t*-tests are one-tailed. Because we had no directional hypotheses for the percent of neurons active during ALT and BAT, all neurobiological IEG *t*-tests are two-tailed.
